# *Babesia microti* Aldo-keto Reductase-Like Protein Involved in Antioxidant and Anti-parasite Response

**DOI:** 10.3389/fmicb.2017.02006

**Published:** 2017-10-11

**Authors:** Qiang Huang, Jie Cao, Yongzhi Zhou, Jingwei Huang, Haiyan Gong, Houshuang Zhang, Xing-Quan Zhu, Jinlin Zhou

**Affiliations:** ^1^Key Laboratory of Animal Parasitology of Ministry of Agriculture, Shanghai Veterinary Research Institute, Chinese Academy of Agricultural Sciences, Shanghai, China; ^2^State Key Laboratory of Veterinary Etiological Biology, Key Laboratory of Veterinary Parasitology of Gansu Province, Lanzhou Veterinary Research Institute, Chinese Academy of Agricultural Sciences, Lanzhou, China; ^3^Jiangsu Co-innovation Center for Prevention and Control of Important Animal Infectious Diseases and Zoonoses, Yangzhou, China

**Keywords:** *Babesia microti*, aldo-keto reductase, antioxidant, drug response

## Abstract

The intraerythrocytic apicomplexan *Babesia microti* is the primary causative agent of human babesiosis, which is an infectious disease that occurs in various regions around the world. Although the aldo-keto reductases (AKRs) of this parasite have been sequenced and annotated, their biological properties remain unknown. AKRs are a superfamily of enzymes with diverse functions in the reduction of aldehydes and ketones. In the present study, we cloned the full-length cDNA of a *B. microti* aldo-keto reductase-like protein (BmAKR) and analyzed the deduced amino acid sequence of the BmAKR protein. This protein has a conserved AKR domain with an N-terminal signal sequence. *Bmakr* was upregulated on the 8th day after infection, whereas it was downregulated during the later stages. The recombinant protein of BmAKR was expressed in a glutathione *S*-transferase-fused soluble form in *Escherichia coli.* Western blot analysis showed that the mouse anti-BmAKR antibody recognized native BmAKR from a parasite lysate. Immunofluorescence microscopy localized BmAKR to the cytoplasm of *B. microti* merozoites in mouse RBCs in this study. *Bmakr* expression was significantly upregulated in the presence of oxidant stress. Atovaquone, a known anti-babesiosis drug, and robenidine, a known anti-coccidiosis drug, induced upregulation of *Bmakr* mRNA, thereby suggesting that *Bmakr* may be involved in anti-parasite drug response.

## Introduction

Babesiosis is caused by about 100 *Babesia* parasite species, and largely affects the cattle industry as well as other companion animals. Some of *Babesia* can affect human, its prevalence in humans has recently gained attention (Smith et al., 2014). It is generally believed that the primary etiological agent is *B. microti* that causes of human babesiosis in the United States, but it is a rare cause of disease in Europe and Asia (Vannier and Krause, 2009; Gabrielli et al., 2016). The second-most important species is *B. divergens* that causes several clinical cases in Europe, and that is the first recognizing pathogeny in a splenectomized patient (Gray, 2006). In china, a recent report describes 48 cases were diagnosed, with *B. venatorum* being the main causative species (Jiang et al., 2015). Other species, such as *B. duncani*, and some *B. divergens*-like parasites, can cause further human infections worldwide (Gray, 2006). Babesiosis is transmitted by the bite of the *ixodid tick*, but may be transmitted also by blood transfusion and transplacentally, and infections usually involve the young and the elderly (Vannier et al., 2015). The clinical presentation includes persistent non-periodic high fever (40–41°C), shaking chills, intense sweats, headaches, myalgia, and lumbar and abdominal pain. Infection usually causes acute onset symptoms that are similar to that of malaria, which include fever, sweating, chills, and fatigue (Gray et al., 2010).

In mammalian hosts, *B. microti* lives in an oxidative stress environment during the erythrocytic stage. It is thus exposed to the toxic effects of ROS, which can damage membrane lipids, nucleic acids, and proteins (Bosch et al., 2015). Thus, to address this challenge and maintain a redox balance, parasites employ a variety of antioxidant systems. Several redox-associated proteins such as thioredoxin (Trx) and peroxiredoxin (Prx) have been demonstrated to be involved in antioxidant systems (Zhang H. et al., 2016; Zhao et al., 2016). AKR is believed to be an important antioxidant component. The members of the AKR superfamily are monomeric cytoplasmic proteins that occur in nearly all phyla, and a nomenclature and precise description of this group of proteins has been made with a specific database^1^ (Jez et al., 1997; Jez and Penning, 2001). These enzymes have broad substrate specificity and transform sugar and lipid aldehydes, keto-steroids, and chemical carcinogens (Penning, 2015). AKRs are regulated by primordial signals such as osmotic shock, ROS, electrophiles, and other environmental stress (Penning and Drury, 2007). Studies in human shows that cell exposure to reactive oxygen species (ROS) can led to a 3- to 10-fold increase in AKR1C Mrna (Burczynski et al., 1999), AKR7A proteins are significantly upregulated in response to acetaminophen/*N*-acetyl-*p*-quinoneimine (APAP/NAPQI) exposure to contribute significantly to protection against APAP-induced hepatotoxicity (Ahmed et al., 2011). Based on its ability to increase intracellular ROS levels and long physiological half-life (8 h–20 days), H_2_O_2_ has been used as a model for oxidative damage (Zhang Y. et al., 2016).

In humans, AKRs have been implicated in the metabolism of synthetic hormones, cancer chemotherapeutics, and CNS-acting drugs (Jin and Penning, 2007), and AKRs is believed to be an important drug target. Quinine and atovaquone are commonly used in the treatment of human babesiosis. Current treatments for human babesiosis consists of two drug combinations, atovaquone + azithromycin or quinine + clindamycin, however, therapeutic regimen are associated with significant side effects and drug failures. Furthermore, the mechanisms by which most of these drugs inhibit intraerythrocytic development of *B. microti* have not been investigated (Lawres et al., 2016). Robenidine is currently only available for the treatment of coccidiosis in chickens and is not licensed for use in humans, but it achieved the best activity against *B. microti* in BALB/c mice (Yao et al., 2015). Robenidine and proguanil (antimalarial drugs) are similar in structure to a guanidine group, but their activity against *Babesia* is different. Artemisinins are the most rapidly acting of currently available antimalarial drugs and is currently used as first-line treatment for *Plasmodium falciparum* worldwide (Dondorp et al., 2010; Woodrow and White, 2016). However, a limited number of reports have confirmed the efficacy of artemisinin derivatives against *Babesia* spp. (Iguchi et al., 2015).

To explore the role of BmAKR in an oxidative environment and the potential of drug target, we cloned and identified the aldo-keto reductase (AKR) of *B. microti* and investigated its response in four agents against babesiosis.

## Materials and Methods

### Parasites and Animals

The *B. microti* strain was obtained from the American Type Culture Collection (ATCC^R^ PRA-99^TM^). Male Slac:KM mice (25 g ± 2 g) were purchased from SLAC Laboratory Animal Co., Ltd. (Shanghai, China), and inoculated intraperitoneally with 0.4 mL of blood containing 1 × 10^8^ erythrocytes of which 20–30% were infected *B. microti*. The percentage of parasitemia was determined by counting the number of parasitemic RBCs in tail blood smears that were stained with Giemsa.

### RNA Isolation and Cloning of the *B. microti* BmAKR Gene

Total RNA was extracted from *B. microti*-infected RBCs (iRBCs) using TRIzol (Invitrogen, Carlsbad, CA, United States) according to the manual’s instructions. cDNAs were obtained by RT-PCR using total RNA as template. The reactions were performed according to the instructions in the ReverTra^®^ Ace qPCR RT Master Mix with gDNA Remover (Toyobo, Osaka, Japan). The potential open reading frame (ORF) of AKR mRNA is present in the genome of *B. microti* (NCBI Reference Sequence: XM_012794167.1) (Cornillot et al., 2012), but the complete sequence of BmAKR cDNA was not available. To identify beginning and end of transcription on the genome we carry out RACE-PCR. We designed oligonucleotide primers from reported sequence to amplify the partial sequence of BmAKR. Their sequences were as follows: F: 5′-TAGCGGAGGAAATGGAGGAAAAAAC-3′ and R: 5′-GATTAATATGTCGCTCCATCATGTCG-3′.

The 5′ and 3′ mRNA termini were amplified, respectively, by using a 5′ rapid amplification of cDNA ends (5′ RACE) and 3′ RACE System for Rapid Amplification of cDNA End Kit (Invitrogen, Carlsbad, CA, United States), following the manufacturer’s instructions. The primers were designed based on the partial sequence of *Bmakr*, which were as follows: GSP1: CTTTTGACACTAACAATCTCTACATAGG, GSP2: GCAAAAATCCGCCTAAGTTGTG, and GSP3: CCTCATGTTTAATGTAGAAGCG for 5′ RACE; and GSP1: ATGATACTAGGAGATCGTCGGGAGA and GSP2: CTAAGCAATGAAACCCCCTGTG for 3′ RACE (Yu et al., 2013; Wang et al., 2015). The PCR products were subsequently purified and linked to a pMD-18T vector (Takara, Dalian, China) and sequenced.

### Expression and Purification of Recombinant Proteins

The ORF of *Bmakr* gene without signal peptide was codon-optimized (**Supplementary Figure S1**) and subcloned into a PUC-57 clone vector, and then subcloned into a pGEX-4T-1 expression vector. The accuracy of the sequences was confirmed by complete sequencing. The BmAKR gene was expressed as a glutathione *S*-transferase (GST)-fusion protein in *Escherichia coli* BL21 (DE3) cells according to the manufacturer’s instructions. The recombinant proteins were induced with 1mM IPTG, followed by incubation at 37°C for 4 h. The resulting *E. coli* cells were washed three times with phosphate-buffered saline (PBS), lysed in PBS containing 1% Triton X-100, sonicated, and then centrifuged at 12,000 × *g* for 10 min at 4°C. Supernatants containing the soluble GST fusion protein were purified with glutathione-Sepharose 4B beads (Amersham Pharmacia Biotech, Inc., United States) according to the manufacturer’s instructions. The purified proteins were dialyzed against PBS for use in subsequent analyses (Zhou et al., 2010; Wang et al., 2015). The empty pGEX-4T-1 vector was used to produce the control GST protein, which was expressed and purified using the same procedure as that for the BmAKR-GST fusion protein. Recombinant protein expression and purification analyses were conducted by standard SDS-PAGE.

### Production of Antiserum against Recombinant BmAKR

Approximately, 100 μg of purified recombinant BmAKR protein was mixed with Freund’s complete adjuvant (Invitrogen) and injected intraperitoneally into mice (KM, 25 g). The 50 μg of purified recombinant BmAKR in Freund’s incomplete adjuvant (Invitrogen) was intraperitoneally injected into the mice on day 14 and again on day 28. Sera from immunized mice were collected 14 days after the last immunization. Serum antibody titers against recombinant BmAKR were measured by ELISA (Wang et al., 2016).

### Western Blot Analysis and Immunofluorescence Assay (IFA)

The lysates of *B. microti*-infected or uninfected mouse erythrocytes were mixed with an equal volume of 2×SDS gel-loading buffer, respectively. Then, the protein samples were electrophoresed on a 10% SDS-polyacrylamide and transferred to an NC membrane by electroblotting. The membranes were blocked in 5% (w/v) non-fat dry milk and incubated with the recombinant BmAKR antiserum (1:200 dilution) at 4°C overnight, followed by three 10-min washes with PBST. Then, the membrane was incubated with HRP-conjugated goat anti-mouse IgG (1:2,000 dilutions) at room temperature for 1 h. After washing with PBST, the proteins of interest were detected by chemiluminescence by using ECL-Plus Blotting Detection System (Bio-Rad, Hercules, CA, United States). Mouse GST-tag monoclonal antibody was used as negative control and was diluted to a ratio of 1:200 (Terkawi et al., 2009). *B. microti*-parasitized erythrocytes were coated on IFAT slides, dried, and fixed in a mixture of methanol and acetone (v:v/1:1) at -20°C for 10 min. After three washes in PBS, the erythrocytes were permeabilized with 0.1% Triton X-100 for 20 min. Then, the fixed smears were incubated with the recombinant BmAKR antiserum (1:100) and incubated for 1 h at 37°C, followed by three 5-min washes in PBS. The slides were then incubated with goat anti-mouse immunoglobulin G conjugated with Alexa 488 (Sigma, St Louis, MO, United States) for 1 h and washed in PBS. Subsequently, the slides were incubated with 4′,6′-diamidino-2-phenylindole (DAPI) (Thermo Scientific, Waltham, MA, United States) for 20 min, washed in PBS, dried, mounted in 10 μL of Fluoromount^TM^ Aqueous Mounting Medium (Sigma), and covered with a glass cover slip. Finally, the slides were examined under a confocal laser scanning microscope (Ooka et al., 2011).

### Expression Analysis of *Bmakr* at Different Days Post-infection

To normalize expression data, β-actin was used as an internal control. The specific primers used to quantify *Bmakr* and *B. microti* actin (Cornillot et al., 2012) (GenBank Accession Number, XM_012793635) were as follows: Forward: CTCAAGGGCTGTGAAAGAGTAC and Reverse: GAAGCTGGTATAAATCGTTGGCC for *Bmakr*, and Forward: GCTCTCATGATTGGAATGGACG and Reverse: AACCGAATGTTCTTCAGGAT for β-actin. The qRT-PCR was performed with a TAKARA SYBR^®^ Premix Ex Taq TM II (Tli RNaseH Plus) (TAKARA, Dalian, China.) using a CFX96 Touch^TM^ Real-Time PCR System (Bio-Rad). Dissociation curve analyses and gel electrophoresis of target gene amplicons were performed for each sample following the qRT-PCR step to ensure primer fidelity. All qRT-PCR amplifications were performed in triplicate and repeated twice, with the mean values considered for comparison. To determine the relative transcriptional level of *Bmakr* at different infection periods, the total RNA extracted from iRBC at different developmental stages were subjected to qRT-PCR analysis. Five groups of mice were injection with 10^8^ iRBCs, and blood was collected from days 2 to 14 after injection.

### Infection of Red Blood Cells, Short-term Culture *in Vitro*, and Measurement of Intracellular ROS

Blood was collected from *B. microti-*infected mice exhibiting 30% parasitemia by heart puncture after anesthesia. The iRBCs were subjected to consecutive centrifugations and washed three times with PBS to remove the buffy coat, plasma, and platelets. A 12-well flat-bottom plate was used for drug screening. A total of 2 × 10^7^ *B. microti* iRBCs were cultured in RPMI without FBS at 37°C in an atmosphere of 95% air and 5% CO_2_ (Fredriksson et al., 2004; Blasa et al., 2011).

The *ex vivo* iRBCs were subjected to short-term treatment with different concentrations H_2_O_2_ (50, 100, and 150 μm), for 30 min, respectively, and then cultured at 37°C and CO_2_. After treatment with H_2_O_2_, the iRBCs were then washed with PBS. The cells were then processed for qRT-PCR and intracellular ROS analyses.

Intracellular ROS production was determined by using 2′,7′-dichlorofluorscin diacetate (DCFH-DA) (Sigma). In principle, DCFH-DA readily penetrates the cell membrane, whereas the diacetate esteric form can be rapidly de-esterified by the membrane-bound enzyme, esterase, to yield the DCFH-free form. The latter is the reduced form of the fluorescent dichlorofluorescein (DCFH). Upon reaction with ROS, DCFH is oxidized to yield a fluorescent DCF, whose intensity can be correlated with the amount of ROS formed *in situ* (Ramful et al., 2010). A stock solution of 10 mM DCFH-DA was prepared in methanol and diluted in PBS to obtain a 20 μM working solution. The iRBC suspension was incubated with 20 μM DCFH-DA at 37°C, and then washed twice in PBS to remove any remaining DCFH-DA in the extracellular medium. The cells were then resuspended in cold PBS and transferred in flow tubes, analyzed with a CytoFLEX flow cytometer (Beckman/Coulter, Fullerton, CA, United States). Fluorescence intensities were measured at an excitation wavelength of 488 nm and an emission wavelength of 530 nm (Blasa et al., 2011). And then, ROS product (fold of control) was calculated as: fold of control = MFI (test)/MFI (control) (He et al., 2014).

### Expression Patterns of *Bmakr* Using Different Anti-parasite Drugs Treatments

To evaluate the effect of metabolic drugs on *Bmakr* gene expression, the short-term *ex vivo* growth of iRBCs exposed to four antiparasitic agents (artemisinin, quinine, robenidine, and atovaquone) was investigated. Artemisinin, robenidine, and atovaquone were dissolved in DMSO, whereas quinine was diluted in ethanol. The iRBCs pretreated with artemisinin, quinine, robenidine, and atovaquone were harvested at 24 h, whereas those pretreated with DMSO or ethanol were used as control. All iRBCs were processed and their *Bmakr* transcript levels were assessed.

### Data Analysis

Data was analyzed by using the Graphpad PRISM 5 software (GraphPad Software Inc., La Jolla, CA, United States). The mean ± standard error (SEM) values of each group were calculated, and two-tailed *t*-tests were used to detect differences among groups. *P* < 0.05 was considered significant and *P* < 0.01 highly significant.

### Animal Care

KM mices were bred and housed in IVC mouse cage with comfortable bedding in the animal facility of Shanghai Veterinary Research Institute. Laboratory rodent food and sterilized water were freely available at all times. Lights were on for 12 h from 06:00 in a 23°C room. We monitor the health of mice every day, basing on apparent inspection such as appetite, responsive, body hair smooth, active, no scars, tail does not bend secretion and excretion. Infected mouse appear apathetic, messy body hair, reduced feeding symptoms in 4–8 DPI, but there were no any unexpected deaths.

### Ethics Statement

The protocols used in this study were approved by the Institutional Animal Care and Use Committee of the Shanghai Veterinary Research Institute and authorized by the Animal Ethical Committee of Shanghai Veterinary Research Institute. Each mouse was sacrificed by cervical dislocation with light anesthesia, and all efforts were made to minimize suffering.

## Results

### Cloning and Sequence Analysis of the Full-Length *Bmakr* cDNA

**FIGURE 1:**
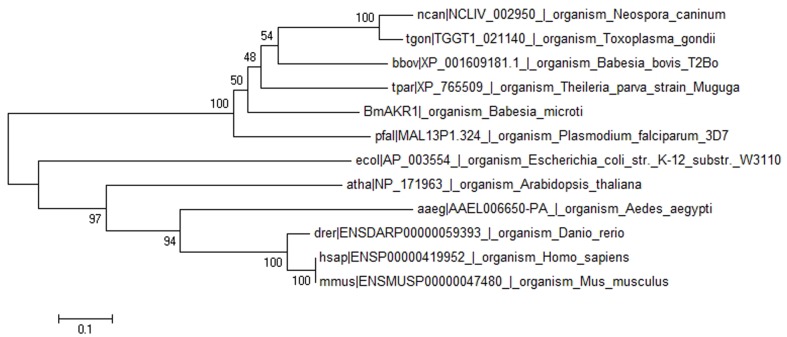
Phylogenetic tree of BmAKR and other species AKR. The rectangular phylogram is based on the alignment of sequences derived from this study using Mega 6.0 by using the maximum likelihood approach.

The full-length *Bmakr* cDNA contained an ORF of 2,145 bp in length, which encoded a 714-amino acid polypeptide (**Figure 1A** and **Supplementary Figure S1**). Its putative protein sequence was analyzed by using SMART^2^ and ExPASy^3^^,^^4^). The *Bmakr* gene included an 8 bp 5′-untranslated region (5′-UTR) before the ATG initiation codon and a 2,142 bp coding sequence terminating with the TAG stop codon, followed by an 81 bp 3′-UTR. There are eight introns in this gene in genome compared with R1 genome sequence (**Figure 1B** and **Supplementary Figure S1**). The results showed conserved domains that were regarded as AKRs. The putative protein has a molecular mass of 83.5 kDa, which is bigger than other AKRs members, and an isoelectric point of 6.26. The N-terminus of the BmAKR protein, particularly between the 19th and 20th amino acids, showed the characteristics of a signal peptide cleavage site (**Supplementary Figure S2**).

BLASTx searches were performed with BmAKR to identify orthologs from other species. The amino acid sequence of BmAKR showed 58.9% similar with *B. bovis* (uniport accession: P40690), 58% sequence similarity with a putative AKR in *Plasmodium falciparum* (NCBI accession XM_001350321.1), 46% with the AKR of *Theileria equi* (NCBI accession XP_004832536), 40% with that of the *Theileria parva* strain Muguga (NCBI accession XP_765509.1). A phylogenetic tree constructed with the complete sequences of BmAKR use relative organism AKR from OrthoMCL (OrthoMCL ID OG5_126648), showed that genetic relationship of BmAKR is far distance from that of other mammalian, but close distance with prokaryotes (**Figure 1**).

### Expression of BmAKR in *E. coli*

**FIGURE 2:**
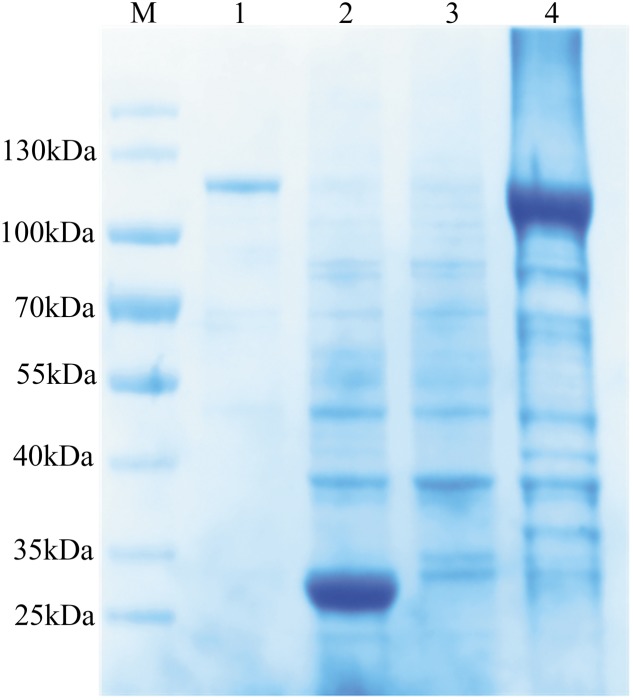
Expression and purification of recombinant BmAKR. Lane 1: purified recombinant protein; Lane 2: The induced lysate of control; Lane 3: The uninduced cell lysate; Lane 4: The induced lysate of recombinant BmAKR. M: Protein molecular weight marker.

The coding sequence of *Bmakr* without the putative signal peptide was subcloned into prokaryotic PGEX-4T-1 expression vectors to generate a recombinant BmAKR. The recombinant protein had a molecular mass of 109 kDa using 12% SDS-PAGE (**Figure 2**), which coincided with the predicted size of the recombinant BmAKR-GST. BmAKR-GST was soluble, was isolated from the supernatant of *E. coli*, and used in the production of polyclonal antibodies.

### Identification of BmAKR Protein in *B. microti*

**FIGURE 3:**
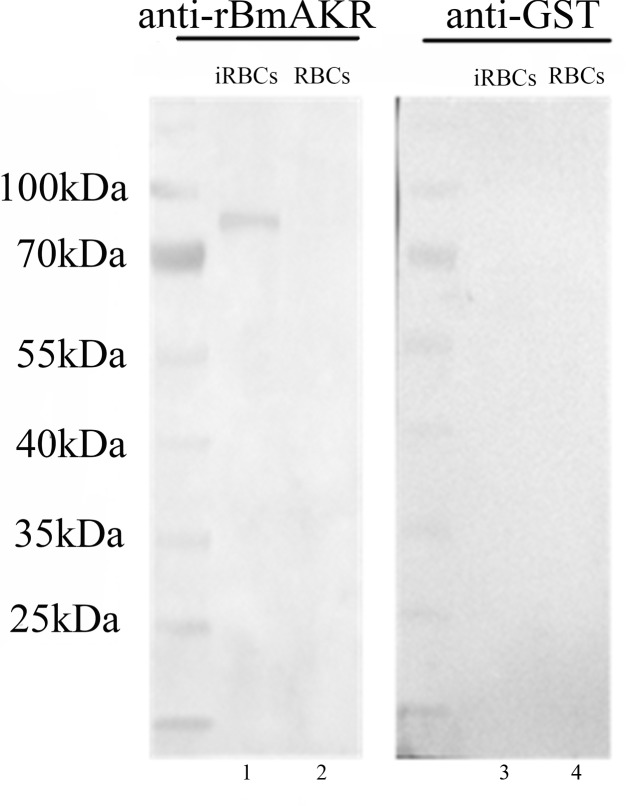
Western blot analysis of BmAKR. Lanes 1 and 3: *Babesia microti*-infected mouse erythrocyte lysate; Lanes 2 and 4: normal mouse erythrocyte lysate: Lanes 1 and 2 were Western blot analysis using an anti-rBmAKR mouse serum; Lanes 3 and 4 was incubated with GST-tag monoclonal antibody. M: protein molecular weight marker.

**FIGURE 4:**
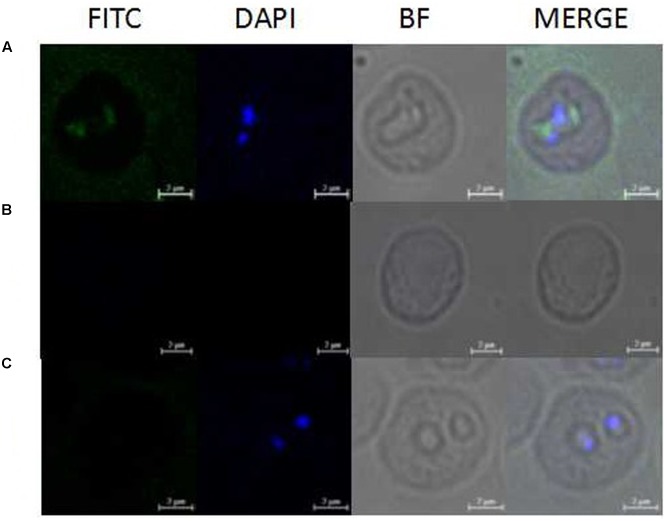
Observation of immunofluorescence microscopy for BmAKR in intraerythrocytic *B. microti* parasites. **(A)** Observation of the BmAKR recognized by a mouse anti-rBmAKR serum in confocal laser micrographs in mouse iRBC. **(B)** Control observation of the mouse iRBC not recognized by a mouse normal serum in confocal laser micrographs. **(C)** Control observation of the normal mouse RBC not recognized by a mouse anti-rBmAKR serum in confocal laser micrographs. FITC, Immunofluorescent staining of *B. microti* merozoites with mouse serum (green); DAPI, 4′,6′-diamidino-2-phenylindole (DAPI) staining of *B. microti* merozoite nuclei (blue); BF, Phase-contrast images of *B. microti* merozoites; MERGE, merged image of FITC, DAPI, and BF; Bars: 2 μm.

Western blot analysis using the antiserum against recombinant BmAKR was used to identify the native BmAKR protein. A specific protein band with a molecular weight of approximately 83.5 kDa was detected in iRBCs but not in the un-infected cells by Western blotting (**Figure 3**). There were no other protein bands recognized by the antiserum, suggested that antiserum against the recombinant BmAKR was specific to the BmAKR protein. These findings demonstrated that *B. microti* expressed BmAKR in its mammalian host. To localize BmAKR in intraerythrocytic *B. microti* parasites, a thin blood smear was prepared for IFA using mouse anti-BmAKR serum and examined by confocal laser microscopy, which showed that BmAKR was located in the cytoplasm of *B. microti* merozoites in mouse RBCs (**Figure 4**).

### Expression Analysis of *Bmakr* at Different Days Post-infection (DPI)

**FIGURE 5:**
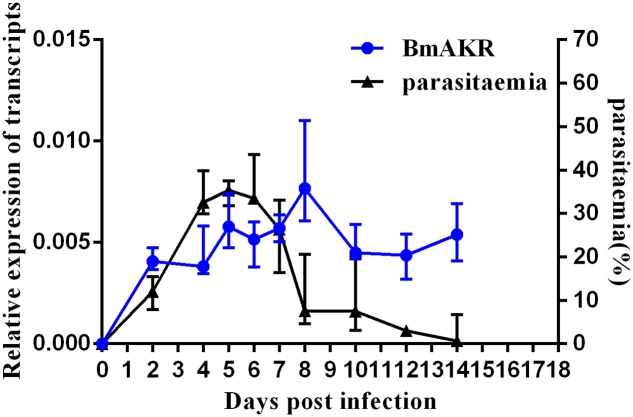
Expression analysis of *Bmakr* at different days post-infection. Expression analysis of *Bmakr* with parasitemia at different days post-infection.

To determine the expression profile of *Bmakr*, total RNA from different days post-infection were subjected to RT-PCR analysis. The progression of infection was examined by counting the number of parasitemic RBCs in the tail blood smears that were stained with Giemsa. *B. microti* was detected in the blood of infected mice at 2 DPI. The percentage of parasitemia progressively increased and peaked (36.5%) at 6 DPI. Subsequently, it declined and reached non-detectable levels by 14 DPI. No *B. microti* was detected in the control mice throughout the experiment. The *Bmakr* mRNA relative expression levels were highest at 8 DPI, which subsequently decreased until the late stationary growth stages (**Figure 5**).

### Oxidant Stress-Induced Expression of the BmAKR Gene

**FIGURE 6:**
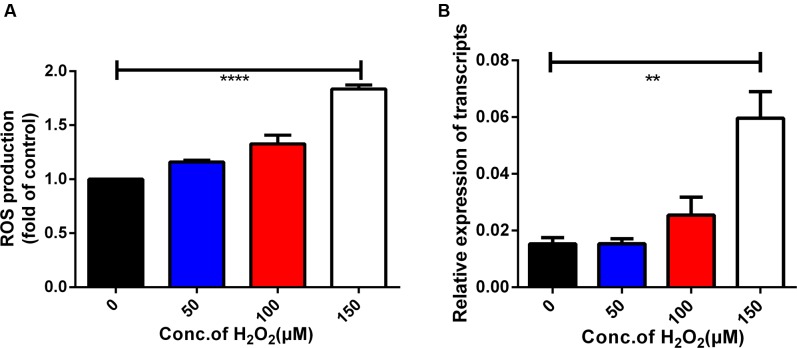
Oxidative stress-induced expression of the *Bmakr* gene. **(A)** Intracellular ROS levels; **(B)** *Bmakr* relative expression of transcripts.

In the present study, iRBCs were treated with H_2_O_2_ to induce excessive ROS production. Intracellular ROS levels in iRBCs were assessed by flow cytometry and correlated with *Bmakr* expression. The DCF fluorescence intensity in iRBCs of the 150 μM H_2_O_2_ group was significantly higher than that of the untreated group (**Figure 6A**), which was indicative of augmented ROS production. Furthermore, *Bmakr* expression was significantly higher than that of the untreated groups (*p* < 0.01), thereby suggesting that H_2_O_2_-induced ROS resulted in the upregulation of *Bmakr* (**Figure 6B**).

### BmAKR Is Implicated in Robenidine and Atovaquone Response

**FIGURE 7:**
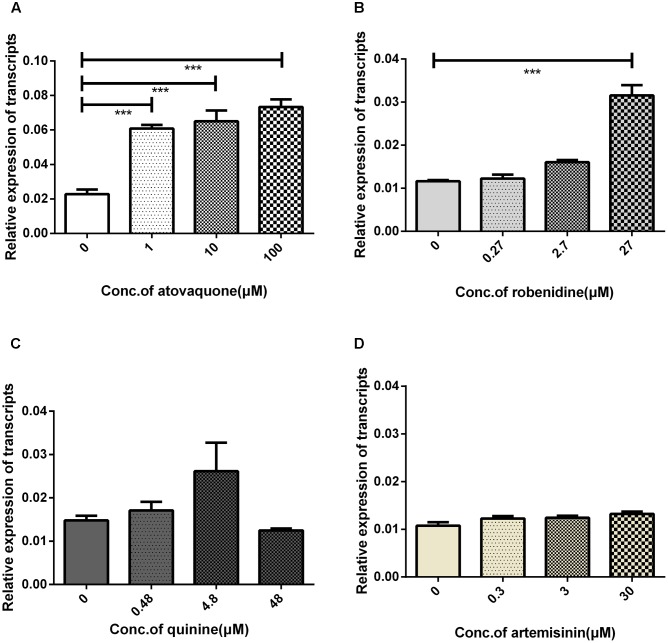
iRBCs exposed to four antiparasitic agents for 24 h. The iRBCs was treated with: **(A)** atovaquone; **(B)** robenidine; **(C)** quinine; **(D)** artemisinin.

*Bmakr* mRNA relative expression levels were analyzed after treatment with the four drugs – robenidine, atovaquone, artemisinin, quinine for 24 h. *Bmakr* relative expression levels in iRBCs were significantly upregulated when exposed to robenidine and atovaquone (**Figures 7A,B**). Furthermore, the robenidine-induced expression of *Bmakr* was is dose-dependent. However, the atovaquone-induced expression of the *Bmakr* showed no discernible differences between the high-dose regimen and the low-dose regimen. The relative expression levels of *Bmakr* showed no significant difference after artemisinin or quinine treatment, respectively (**Figures 7C,D**). These findings indicate that BmAKR may implicate in the response of robenidine and atovaquone.

## Discussion

Aldo-keto reductases exist in nearly all phyla and are mainly NAD(P)(H)-dependent oxidoreductases. Over the last few years, AKRs have been identified and annotated in *B. bovis* (Dalrymple et al., 1992), *T. cruzi* (Garavaglia et al., 2010), and *L. donovani* (Rath et al., 2009). In the present study, we described the molecular cloning and biochemical characterization of AKR of *B. microti*. This enzyme forms a distinct and distant clade from mammalian, plant, and prokaryotic AKRs, which demonstrates that it is a novel enzyme. Previous investigations have shown that AKR is an obligatory mediator of oxidative stress-induced inflammatory pathologies such as diabetes, asthma, sepsis, and cancer, and suggest that the inhibition of AKR could prevent cancer, which validates the protein as an excellent drug target (Ramana and Srivastava, 2010; Tammali et al., 2011).

Recombinant BmAKR was expressed as a GST fusion protein in *E. coli*, and after purification by GST chromatography, polyclonal antibodies were produced using recombinant BmAKR, and the native BmAKR in *B. microti* was detected by Western blotting using its antiserum. IFA showed that BmAKR co-localized with the cytoplasm of *B. microti* merozoites in mouse RBCs in this study. In mammals, AKRs are found in the cytosol (Weber et al., 2015), mitochondria (Weber et al., 2015), and cytomembrane (Xie et al., 2011). In *B. microti*, BmAKR was also localized to the plasma of the parasite. Changes in the progression of infection in KM mice were in agreement with those of previous research investigations (Chen et al., 2000). Expression analysis of *Bmakr* at different DPIs showed the highest expression at 8 DPI; the parasites were eliminated rapidly to a low infection level from 6 to 8 DPI by the immune response of host. Host immune response generates reactive oxygen and nitrogen species (ROS, RNS) to combat the infection (Becker et al., 2004; Zhao et al., 2016). We deduced that *B. microti* promotes *Bmakr* expression to counteract the oxidative stress. The level of *Bmakr* mRNA expression in iRBCs was three-fold higher after treatment with 150 μm H_2_O_2_ relative to that of normal iRBCs. AKR is regulated by a variety of intracellular and environmental signals, including ROS, electrophiles, and chemopreventive agents (Ahmed et al., 2011) and is catalytically active toward aldehydes that arise from lipid peroxidation, thereby suggesting its potential role in combating oxidative stress and representing an important detoxification route in mammalian cells (Li et al., 2012). Oxidative stress-induced *Bmakr* upregulation thus represents a protective response against intracellular ROS, thereby confirming that *B. microti* promotes *Bmakr* expression to counteract the resulting oxidative stress that is induced as part of the host’s Immune response.

In *Trypanosoma cruzi*, AKRs use quinone and benznidazole as a substrate, thereby indicating that this enzyme participates in the mechanism of action of trypanocidal drugs (Garavaglia et al., 2010). In *Schistosoma japonicum*, a lead compound, which inhibited enzymatic activity of recombinant *S. japonicum* AKR but showed relatively low cytotoxicity against host cells, and it suggested that it may be potentially used in the treatment of Schistosomiasis (Liu et al., 2013). A recent study identified variants in cytb and rpl4 that were associate with relapsing babesiosis (Lemieux et al., 2016), but parasites often utilize various mechanisms for drug resistance (Garcia-Salcedo et al., 2016; White, 2017). Therefore, to understand the role of BmAKR in the mechanism of action of anti-*Babesia* drugs and to improve chemotherapy of babesiosis, we studied *Bmakr* expression when *B. microti* is exposed to atovaquone, robenidine, quinine, and artemisinin. Robenidine and atovaquone promote *Bmakr* transcription, which indicates that it is involved in the mechanisms of action of these drugs, as well as those of membrane transporters or other physiological process. Research investigations that would further explore this interesting phenomenon are thus warranted. Besides, due to its distantly related to mammalian AKRs, BmAKR may have great value for the study of a drug target against *B. microti*.

## Author Contributions

JZ conceived and designed the study, and critically revised the manuscript. QH performed the experiment, analyzed the transcriptomic data, and drafted the manuscript. YZ and JC participated in the animal experiments. JH performed and analyzed the results of the confocal laser microscopy observation. HG and HZ participated in the experiments and in data interpretation. X-QZ participated in project planning and manuscript revision. All authors read and approved the final manuscript.

## Conflict of Interest Statement

The authors declare that the research was conducted in the absence of any commercial or financial relationships that could be construed as a potential conflict of interest.

## Footnotes

1 https://www.med.upenn.edu/akr

2 http://smart.embl-heidelberg.de

3 http://web.expasy.org/computepi/

4 http://prosite.expasy.org/

## Supplementary Material

**FIGURE S1 |** Nucleotide sequence with deduced amino acid sequence and genome structure of *Bmakr*. **(A)** The putative signal peptide is underlined. **(B)** Sketch map of *Bmakr* genome structure.

**FIGURE S2 |** Putative signal peptide. The signal peptide analyze of BmAKR by signalIP.
