# Supplementary figures and images for: Babesia microti Aldo-keto Reductase-Like Protein Involved in Antioxidant and Anti-parasite Response
Source: Front Microbiol. 2017 Oct 11;8:2006. doi: 10.3389/fmicb.2017.02006 (PMC5641555; doi:10.3389/fmicb.2017.02006)

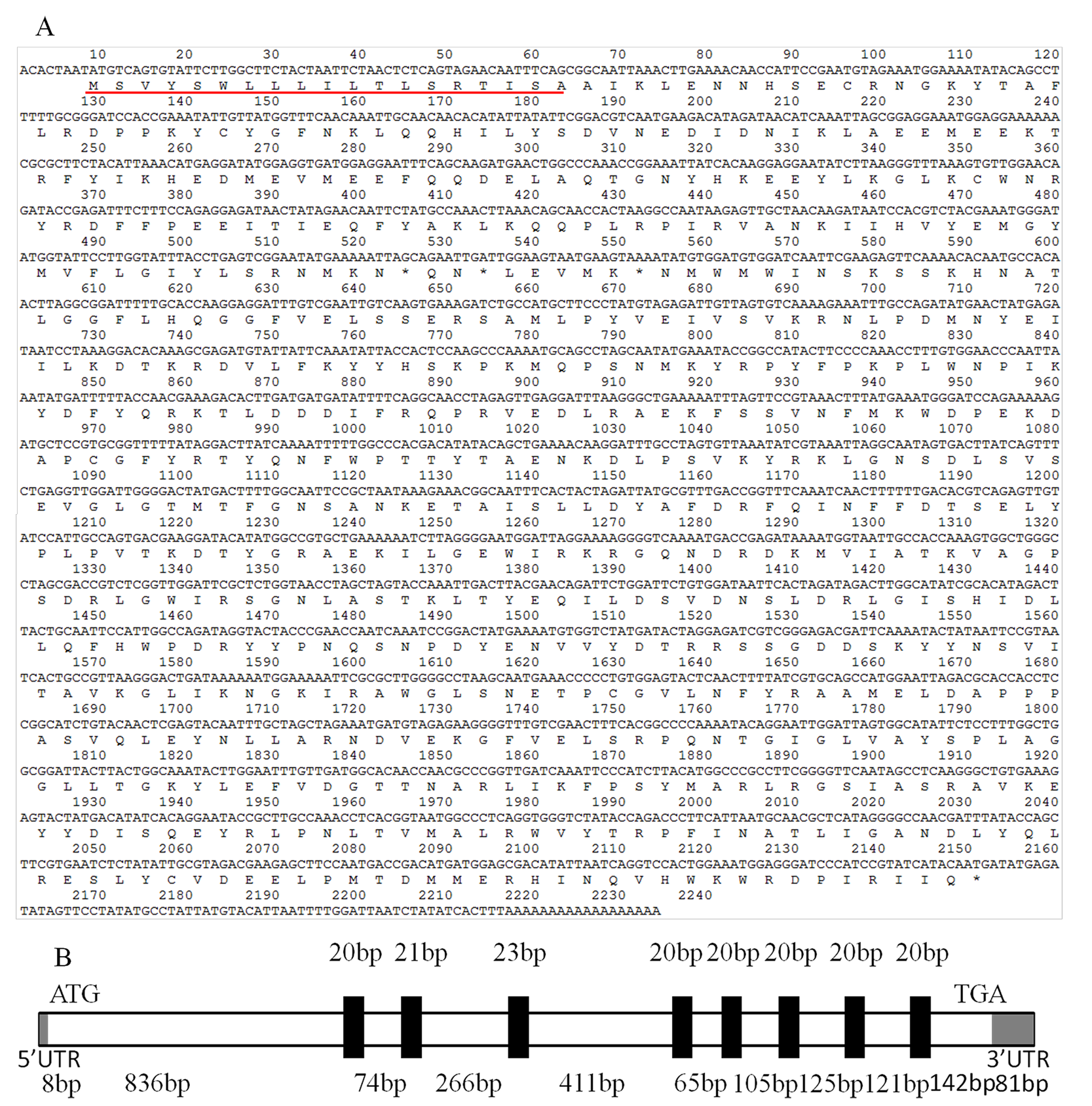

Supplement: Supplementary file 1 [file Image_1.TIF]

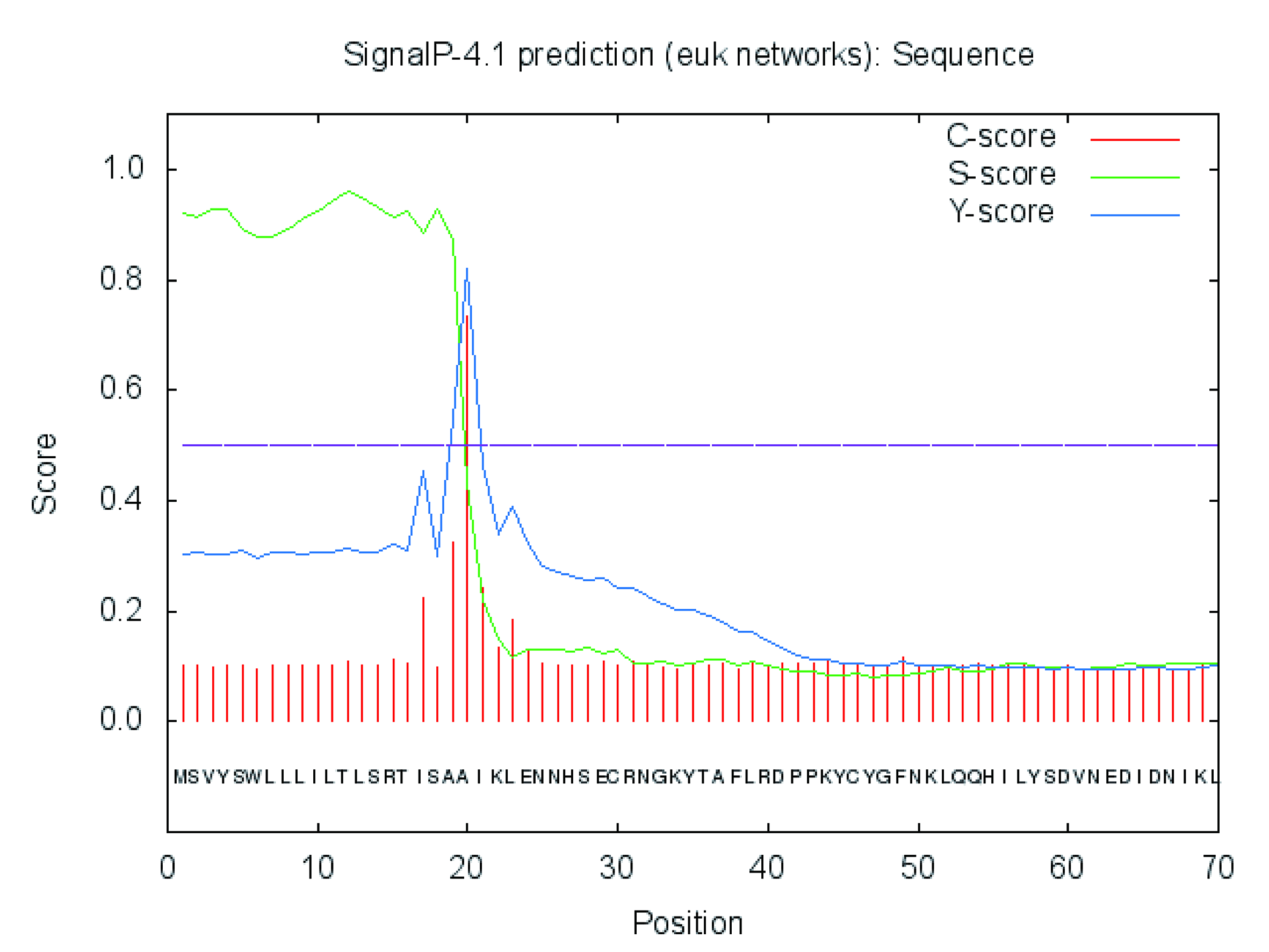

Supplement: Supplementary file 2 [file Image_2.TIF]
